# The Establishment and Characteristics of Rat Model of Atherosclerosis Induced by Hyperuricemia

**DOI:** 10.1155/2016/1365257

**Published:** 2015-12-21

**Authors:** Zhen Liu, Tong Chen, Haitao Niu, Wei Ren, Xinde Li, Lingling Cui, Changgui Li

**Affiliations:** ^1^Shandong Provincial Key Laboratory of Metabolic Disease, The Affiliated Hospital of Qingdao University, Qingdao 266003, China; ^2^Shandong Gout Clinical Medical Center, Qingdao 266003, China; ^3^Key Laboratory of Hypertension, Qingdao 266003, China; ^4^Department of Urology, The Affiliated Hospital of Qingdao University, Qingdao 266003, China

## Abstract

Epidemiological studies have identified hyperuricemia as an independent risk factor for cardiovascular disease. However, the mechanism whereby hyperuricemia causes atherosclerosis remains unclear. The objective of the study was to establish a new rat model of hyperuricemia-induced atherosclerosis. Wistar-Kyoto rats were randomly allocated to either a normal diet (ND), high-fat diet (HFD), or high-adenine diet (HAD), followed by sacrifice 4, 8, or 12 weeks later. Serum uric acid and lipid levels were analyzed, pathologic changes in the aorta were observed by hematoxylin and eosin staining, and mRNA expression was evaluated by quantitative real-time polymerase chain reaction. Serum uric acid and TC were significantly increased in the HAD group at 4 weeks compared with the ND group, but there was no significant difference in serum uric acid between the ND and HFD groups. Aorta calcification occurred earlier and was more severe in the HAD group, compared with the HFD group. Proliferating cell nuclear antigen, monocyte chemotactic factor-1, intercellular adhesion molecule-1, and vascular cell adhesion molecule-1 mRNA levels were increased in the HFD and HAD groups compared with the ND group. This new animal model will be a useful tool for investigating the mechanisms responsible for hyperuricemia-induced atherosclerosis.

## 1. Introduction

Uric acid is an end product of purine metabolism in humans and is excreted in the urine. Loss of uricase function means that humans and other primates have relatively higher levels of serum uric acid compared with rodents, providing the biochemical bases for the inflammatory response in gout and an increased risk of cardiovascular disease. Gertler et al. [1] initially proposed the existence of a complex interaction between uric acid and coronary heart disease in 1951; since then increasing numbers of studies have confirmed a link between raised serum uric acid levels and cardiovascular events. A recent meta-analysis of prospective studies showed that each additional 1 mg/dL of serum uric acid equated to a 12% increase in mortality for patients with coronary heart disease [2]. Raised serum uric acid levels are associated with approximately 70% increase in the risk of coronary heart disease. In addition to its direct cardiovascular effects, hyperuricemia has also been associated with increased risks for the development of hypertension, renal damage, and metabolic syndrome, which indirectly lead to the occurrence of cardiovascular events or affect the prognosis and therapy of cardiovascular disease.

However, conversely, some researchers suggest that the lack of uricase is an evolutionary advantage for primates [3, 4]. Hyperuricemia could help to stabilize blood pressure, and uric acid has antioxidant activities [3, 5, 6]. Hink et al. [7] reported that uric acid could also prevent the degradation of extracellular superoxide dismutase 3, which is a key enzyme for maintaining the functions of endothelial cells and blood vessels. Increased serum uric acid in patients with cardiovascular disease may be an important compensatory mechanism for oxidative stress during the course of the disease [8]. Hyperuricemia was associated with better prognoses in patients with stroke or other neurological disorders [9]. However, these observations fail to explain why higher uric acid levels are associated with a poorer prognosis in patients with cardiovascular disease. The validity of hyperuricemia as an independent risk factor for cardiovascular disease thus remains controversial.

In this study, we established an animal model to investigate the association between hyperuricemia and atherosclerosis risk and also examined the specific molecules involved in this process. The results have important implications for future clinical treatment strategies and for the early prevention of hyperuricemia and atherosclerosis.

## 2. Materials and Methods

### 2.1. Animal Model

Ninety male Wistar-Kyoto rats were purchased from the Animal Center of Beijing University, Beijing, China. Animal experiments were performed in accordance with the guidelines for the Principles of Laboratory Animal Care and the Guide for Care and Use of Laboratory Animals. Rats (200–220 g) were randomly divided into three groups fed a normal diet (ND; *n* = 30), high-fat diet (HFD; *n* = 30), or high-adenine diet (HAD; *n* = 30), respectively. HFD rats were administered intragastric (i.g.) vitamin D_3_ (60 IU/kg) for 3 days followed by a dose of 5 mL/kg high-fat emulsion containing pyrimidine (200 g pork, 200 g cholesterol, 20 g bile salts, and 10 g propylthiouracil, dissolved in 1 L distilled water) twice daily, by intragastric administration. HAD rats were fed with fodder containing 10% yeast powder and administered adenine (50 mg/kg, i.g.) and potassium oxonate (100 mg/kg) subcutaneously twice a day (8 am and 4 pm). ND rats were administered an equal volume of normal saline i.g. and fed a normal diet. The rats were housed individually in specific pathogen-free conditions at a constant temperature (20–22°C) and humidity (45–55%) with a 12 h light-dark cycle. Rats from the three groups were sacrificed after 4, 8, and 12 weeks, respectively.

### 2.2. Serum Uric Acid and Lipid Measurements

At the end of the experimental periods, rats were fasted for at least 8 h and then anesthetized with 10% chloral hydrate. Blood samples were obtained from the right carotid artery after 4 h at room temperature and centrifuged at 3500 rpm for 15 min at room temperature. Serum was separated and uric acid and lipid levels were determined using an autoanalyzer (Toshiba, Japan).

### 2.3. Tissue Processing, Histology, and Immunohistochemistry

At the end of the experiment, the thoracic aorta (from the arch to the diaphragm) was harvested, cut in half, and either fixed in buffered formalin or snap frozen. Aorta rings were embedded in paraffin and sections were cut at 4 *μ*m and prepared for hematoxylin and eosin (HE) staining. Immunohistochemical detection of proliferating cell nuclear antigen (PCNA) was performed using an anti-PCNA primary antibody (1 : 500) and a horseradish peroxidase-conjugated goat anti-rat Ig secondary antibody (1 : 100) (Invitrogen, Basel, Switzerland).

### 2.4. Quantitative Real-Time Polymerase Chain Reaction

Total aorta RNA was extracted using Trizol reagent (Invitrogen). For each sample, cDNA was synthesized using a PrimeScript RT Reagent Kit (Takara, China) according to the manufacturer's instructions. SYBR Green-based polymerase chain reaction (PCR) was performed in an automated thermal cycler (Bio-Rad) in a final volume of 25 *μ*L, containing 2 *μ*L cDNA solution, 12.5 *μ*L of SYBR Premix Ex Taq (Takara), 1 *μ*L of each primer (10 *μ*mol/L), and 8.5 *μ*L of ddH_2_O. The cycling reaction was performed according to the manufacturer's instructions via a standard two-step PCR. Experimental Ct values were normalized to *β*-actin and relative mRNA expression was calculated in comparison with a reference sample. Each sample was run and analyzed in triplicate. The structures of the primers used are listed in Table 1.

### 2.5. Statistical Analyses

All data are expressed as mean ± standard deviation and were analyzed by one-way analysis of variance followed by Newman-Keuls multiple comparison test as appropriate (GraphPad Prism version 5 software). A value of *P* < 0.05 was considered statistically significant.

## 3. Results

### 3.1. Changes in Serum Uric Acid and Lipid Levels

**Figure 1:**
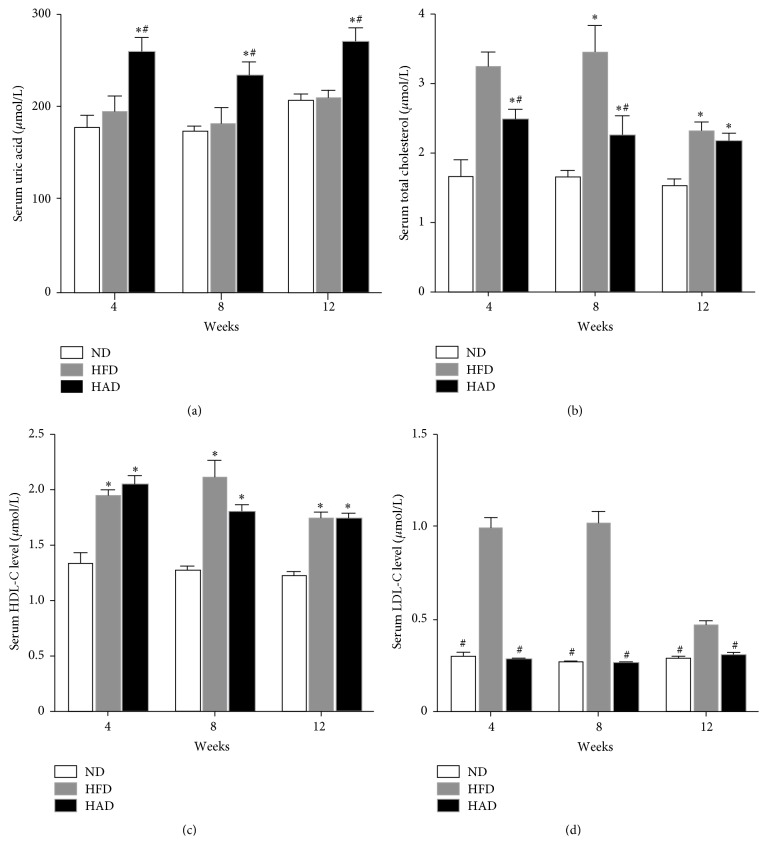
Serum uric acid and lipid levels in rats. Serum uric acid and lipid levels were analyzed using an autoanalyzer. (a) Serum uric acid, (b) serum TC, (c) serum HDL-C, and (d) serum LDL-C (*n* = 8–10). ^*∗*^ *P* < 0.05, compared with the ND group; ^#^ *P* < 0.05, compared with the HFD group.

Serum uric acid and lipid levels were measured in all groups. Serum total cholesterol (TC), high-density lipoprotein cholesterol (HDL-C), and low-density lipoprotein cholesterol (LDL-C) were elevated in the HFD group compared with the ND group after 4, 8, and 12 weeks, respectively, while levels of UA were unchanged. However, serum levels of UA, TC, and HDL-C were increased in the HAD rats. Interestingly, TC levels were lower in the HAD compared with the HFD group (Figure 1). Furthermore, mortality was increased by 30% in the HAD group after 8 weeks, compared with the ND and HAD groups.

### 3.2. Histopathological Changes in Aortas

**Figure 2:**
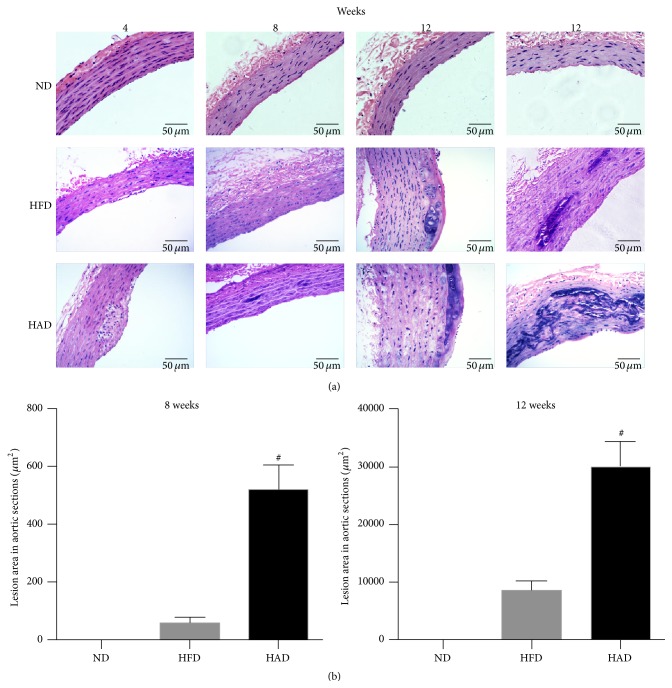
Histopathological examination of aorta in rats. All rats were sacrificed at 4, 8, or 12 weeks, respectively. (a) Aorta rings were stained with HE (×400 magnification). (b) Quantitation of lesioned area in HE-stained aorta sections using Image-Pro Plus software (*n* = 6). ^#^ *P* < 0.05, compared with the HFD group.

There were no pathologic changes in the aortas of ND rats during the course of the experiment. However, foam cells were observed following mononuclear cell infiltration in the aortas of HFD and HAD rats after 4 weeks of treatment. After 8 weeks, numerous foam cells were formed and nuclear condensation appeared in medial smooth muscle cells in HFD rats, while calcium deposits were found in the aortas in HAD rats at 8 weeks and were more severe at 12 weeks. However, no calcium deposits were detected in the aortas of HFD rats up to 12 weeks. Hyaline degeneration occurred in both HFD and HAD rats after 12 weeks' treatment (Figure 2).

### 3.3. PCNA Expression in Aortas

**Figure 3:**
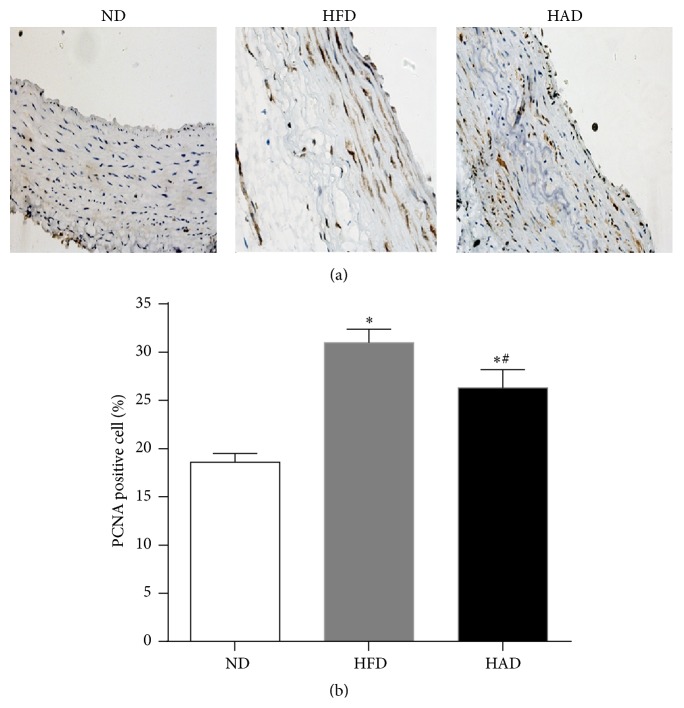
Immunohistochemical staining of aorta in rats. (a) Immunohistochemical staining for PCNA in the aorta (×400 magnification). (b) PCNA protein levels were quantified in each group (*n* = 6). ^*∗*^ *P* < 0.05, compared with the ND group; ^#^ *P* < 0.05, compared with the HFD group.

PCNA expression in the aorta was examined by immunohistochemistry. Staining was primarily located in the medial smooth muscle cells (Figure 3) and was significantly increased in HFD compared with HAD rats (26.3 ± 1.9 versus 31.4 ± 1.4, *P* < 0.05).

### 3.4. Monocyte Chemotactic Factor-1, Intercellular Adhesion Molecule-1, and Vascular Cell Adhesion Molecule-1 mRNA Expression in Aortas

Atherosclerosis in HAD rats was characterized by marked increases in aortic monocyte chemotactic factor-1 (MCP-1), intercellular adhesion molecule-1 (ICAM-1), and vascular cell adhesion molecule-1 (VCAM-1) mRNA compared with the ND group, according to real-time PCR. However, upregulation of MCP-1 was lower in the HAD than in the HFD group.

## 4. Discussion

Hyperuricemia (defined as ≥7.0 mg/dL in male individuals and ≥6.0 mg/dL in female individuals) is caused by increased synthesis and/or reduced excretion of uric acid. The regulation of uric acid is complex, and hyperuricemia is also related to the development of multiple complications, including gout, hyperlipidemia, hypertension, and cardiovascular disease [10–13]. In the current study, we aimed to establish an animal model of hyperuricemia-induced atherosclerosis by feeding rats HAD. This resulted in significant increases in uric acid, TC, inflammation, and calcification, which are known to be associated with atherosclerosis, suggesting that this model may help to further our understanding of the mechanisms whereby hyperuricemia induces atherosclerosis.

Rodents possess uricase, which metabolizes uric acid, and serum uric acid levels are therefore low. Further investigation of the relationship between hyperuricemia and atherosclerosis thus requires a hyperuricemic animal model. We created a rat model by feeding with a mixed diet of adenine, yeast power, and potassium oxonate. After 4 weeks, HAD rats had high serum TC levels. Dyslipidemia is important biochemical basis of atherosclerosis, and lipid accumulation both within and outside cells is a pathological feature of atherosclerotic plaques. Our results suggest that uric acid may interfere with lipid metabolism in rats resulting in abnormal lipid levels, which may provide a mechanism for hyperuricemia-induced atherosclerosis. HE staining showed that vascular smooth muscle cells (VSMCs) were damaged and ultimately disappeared in HAD rats after 4 weeks, with the appearance of foam cells. Furthermore, a high uric acid diet for 8 weeks resulted in diffuse deposits of calcium salts in the medial layer of the vasculature, damage to and fibrosis of subintimal smooth muscle cells, hyaline degeneration, and the appearance of foam cells. Notably, pathologic changes characteristic of atherosclerosis appeared sooner and became more severe in HAD compared with HFD rats.

VSMCs are one of the major cell types involved in atherosclerotic-plaque formation, and excessive proliferation of VSMCs is an important pathological characteristic of atherosclerosis. Soluble uric acid has been shown to cause proliferation of VSMCs [14]. During the early stage of atherosclerosis, medial smooth muscle cells undergo excessive proliferation and migrate to the intima, resulting in thickening of the intima and narrowing of the lumen. Transformation, proliferation, and migration of VSMCs are the basic pathological processes responsible for luminal stenosis [15, 16]. VSMCs are also important source of cytokines and play major role in maintaining and amplifying inflammatory proliferation. The number of PCNA-positive cells can thus reflect the status of cell proliferation [16] and provides important indicator for judging the biological activity and growth status of cells. In the current study, the surface area and thickness of the vessels (data not shown) and the ratio of PCNA-positive cells increased significantly in high uric acid rats. This result further confirmed the role of high uric acid levels in the proliferation of smooth muscle cells, which in turn mediate medial vascular sclerosis and the development of atherosclerosis.

**Figure 4:**
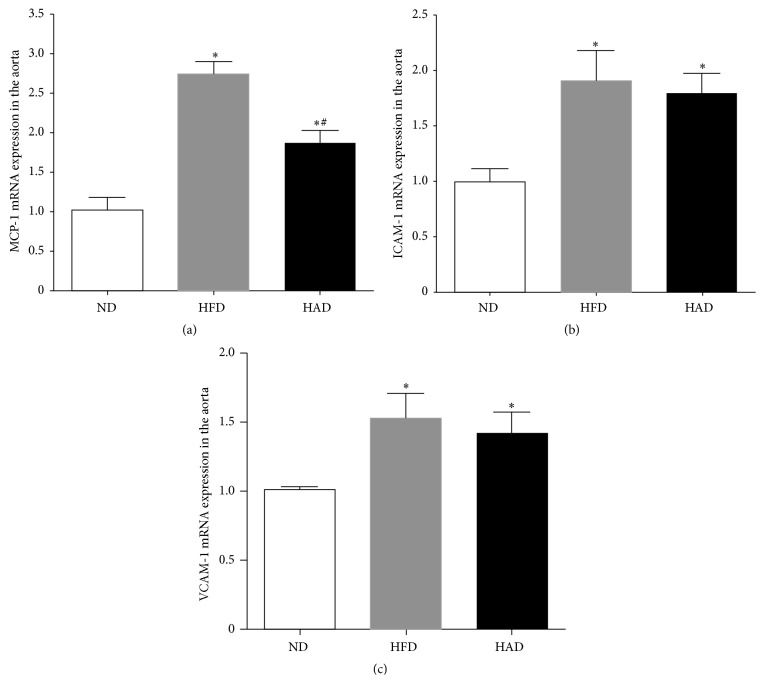
Gene expression in aorta of rats. Gene expression was measured by real-time PCR in each group. (a) MCP-1 mRNA, (b) ICAM-1 mRNA, and (c) VCAM-1 mRNA (*n* = 8–10). ^*∗*^ *P* < 0.05, compared with the ND group; ^#^ *P* < 0.05, compared with the HFD group.

We investigated the possible mechanisms of hyperuricemia-induced atherosclerosis in these model rats. Inflammation represents important mechanism for atherosclerosis, and uric acid is known to have a proinflammatory effect on vascular cells. In smooth muscle cells, high levels of uric acid induced MCP-1 by activating the transcription factor nuclear factor *κ*-B, mitogen-activated protein kinases, and cyclooxygenase 2, while expression levels of ICAM-1, VCAM-1, P-selectin, and E-selectin were also increased. We demonstrated that the mRNA expression levels of MCP-1, ICAM-1, and VCAM-1 in the aorta were increased in HAD rats compared with control rats, based on reverse-transcription PCR (Figure 4). Only upregulation of MCP-1 was lower in the HAD compared with the HFD group. These results indicate that high uric acid levels caused vascular damage and the development of atherosclerosis through upregulation of MCP-1, ICAM-1, and VCAM-1 mRNAs.

## 5. Conclusions

We established an animal model of hyperuricemia-induced atherosclerosis and found no significant difference between hyperuricemia and hypercholesterolemia in causing atherosclerosis in rats. High uric acid levels may cause atherosclerosis via disturbing lipid metabolism, promoting the proliferation of VSMCs, and by activation of inflammation. Hyperuricemia should therefore be regarded as an important risk factor for the occurrence and development of atherosclerosis, and the treatment of hyperuricemia represents an important approach to its prevention and treatment.

## Figures and Tables

**Table 1:** Sequences of primers.

Gene name	Product size	Sense primer	Antisense primer
Rat MCP-1	120 bp	TGTCCCAAAGAAGCTGTAGTATTTGT	TTCTGATCTCACTTGGTTCTGGTC
Rat ICAM-1	103 bp	GGTGGGC AAGAACCTCATCCT	CTGGCGGCT CAGTGTCTCATT
Rat VCAM-1	110 bp	CGAAAG GCCCAGTTG AAG GA	GAGCACGAGAAGCTCAGGAGA AA
Rat *β*-actin	120 bp	TGG ACA TCC GCA AAG AC	GAA AGG GTG TAA CGC AACTA
